# The Modal Components of Judgements in a Quantum Model of Psychoanalytic Theory

**DOI:** 10.3390/e25071057

**Published:** 2023-07-13

**Authors:** Giulia Battilotti, Miloš Borozan, Rosapia Lauro Grotto

**Affiliations:** 1 Department of Mathematics, University of Padua, 35122 Padova, Italy; 2 Department of Neurosciences, Imaging and Clinical Sciences, University of Chieti-Pescara, 66100 Chieti, Italy; milos.borozan@gmail.com; 3 Center for Advanced Studies and Technology (C.A.S.T.), University of Chieti-Pescara, 66100 Chieti, Italy; 4 Department of Health Sciences, University of Florence, 50121 Firenze, Italy

**Keywords:** psychoanalytic theory, affective quantum cognition, abstraction, modal operator, duality and symmetry, infinite singleton, spin observables, Pauli matrices

## Abstract

In the present paper, we develop a theory of thinking based on an attempt to formalize the construction of mental representations as described in psychoanalytic theory. In previous work, we described Freud’s and Matte Blanco’s structural Unconscious in a formal model in which the properties of unconscious representations are captured by particular sets-infinite singletons-that can be derived in first-order logic language. Here, we afford the issue of the finitization of unconscious representations by assuming that the mind can form an all-purpose modality, originating from abstraction from infinite singletons; in this way, a symmetric prelogical setting for mental representations is formally created, and this is interpreted in a quantum spin model by a modal (necessity) projector. Then, by introducing time, one can describe the links that mental representations can establish with reality, and hence finitize the representations. The modality is so split into finite components, here termed positive, negative and irreal; the splitting of the modality is traced back to the decomposition of the spin observables by means of the Pauli matrices, which can offer a quantum semantics to the method applied. Here, we suggest that the development of the modal approach and its quantum logic implementation can be considered as a proper formalization of some aspect of the psychoanalytic theory of thinking proposed by Bion; namely, we will show that the process of abstraction leading from raw data to preconceptions, and therefore to the definition of the content-container relationship, is adequately captured by our model, and further correspondences can be detected with Bion’s theory about links and transformations, implying different ways in which the mind can get in touch with both internal and external reality.

## 1. Introduction

Quantum cognition has been a major challenge in recent studies. Here, we develop a logical basis for a particular approach to quantum cognition, which we term affective quantum cognition. According to psychoanalysis, the unifying dimension of affect has a central role in the representational domain, while in the cognitive sciences, multiple psychological dimensions are considered in order to describe the emotional and affective content of mental life, such as feelings, emotions, sensations and so on, and these dimensions are mainly considered to be separate, distant from cognitive contents [1]. The effort to formalize Freudian theory dates back to the pioneering work of A. Khrennikov, who was the first to recognize a well-defined topological structure in the Freudian Unconscious (see [2,3,4]). Recently, such models were found to be be suitable also to the domain of quantum cognition (see [5,6,7], in the framework of so-called computational psychoanalysis).

In our search, we noted that to think both of the quantum world and of the inner mental world, one has to refer to a common starting point: the indefinite, which means, in different terms, the nonseparability of objects. It is nonetheless a difficult starting point, since in order to formulate a model and communicate it, one needs a language, and language, by its own nature, defines. Ipso facto, this creates a deep impact on the mind, forcing it towards the finite and the determined, as already stressed in *The Violence of Interpretation: from Pictogram to Statement*, by the French psychoanalyst P. Aulagnier [8] and by Freud in [9]. We would like to recall that a similar kind of critique of language in general, and specifically of *formal* language, was originally expressed by the intuitionistic school in mathematics (see [10]). However, shortly after, intuitionism had to surrender to the necessity of communication and adopt a formal language. Namely, the one based on first-order language and the use of logical terms formed from constants and variables in formulae constructed by dual pairs of connectives, as in classical logic.

In order to model Bi-logic, introduced by the Chilean psychoanalyst I. Matte Blanco [11], we found a way to tackle the issue of first-order language via the notion of *infinite singleton*, namely a particular kind of domain for logical quantifiers (see [12]). In turn, this setting has been derived from a model of quantum states in first-order language ([13]). Infinite singletons are a good starting point to approach the indefinite, since bi-logic consists of two modes that are characterized by their opposite attitude with respect to the separation of objects: one, the symmetric mode, works on the infinite, homogeneous and indivisible; the other, the bivalent mode, works on the finite, heterogeneous and separable.

Logic (at least formal logic) works mostly on the bivalent mode, since it is usually studied as a modular object, namely as something composed from elementary statements by means of logical connectives, whereas the attention given to the nature of the elementary statements, their origin and the role they can have in the formation of the logical connectives themselves is usually very low. In order to change perspective, the first step is that of considering and then eventually untangling an aggregate that is created altogether at first, rather than considering how to put statements together when they are assumed to be already and singularly defined.

The main proposal of the paper is that our mind can form an all-purpose modality, named a *modal* projector, originating from abstraction from infinite singletons. The latter are derived by a particular reading of first-order language that can represent Freud’s and Matte Blanco’s structural Unconscious. Abstraction allows to take advantage of the psychoanalytic approach to the Bion’s theory of thinking in the terms of content–container relationship (see below). Overall, a symmetric prelogical setting for mental representations is created that is interpretable in an abstract space by the modal projector. Then, by introducing time, one can describe the links that mental representations can establish with reality, and hence finitize the representations. The modality is so split into finite components, here termed positive, negative and irreal. The last is a *psychotic* component, as interpreted by Bion, given by the failure of the contact with reality: the rational way out, for the mind, is the possibility of dual connectives, and hence the arising of a logical setting. The splitting of the modality is traced back to the decomposition of the spin observables by means of the Pauli matrices. In particular, we see how the original prelogical stage can be kept as an essential infinite component of judgement.

Then, the semantics for our approach is offered on one side by the quantum spin model, and on the other by Freudian theory in its various aspects. We consider, beyond Matte Blanco’s Bi-logic, the main points proposed by Freud from his original theory of representation [14], his characterization of thinking in terms of primary and secondary process in *The interpretation of dreams* [15], his introduction of the tripartite conception of the psychic apparatus (in [16]) and his interpretation of negation and judgements [17]. In addition, the theory of Bion [18] allows us to find the correspondence of the modal projector with his idea of preconception and then with the container–contained relationship (see Section 6), which in turn creates a positive and a negative link of mental representations and external reality. Here, the positive link entails the possibility to develop mental representations, and therefore to experience both mental life and external reality, while the negative links entail an impossibility in doing so [19]. So, the positive link is related to the mature aspects of personality, which develops by remaining in touch with reality, while the negative one is related to the psychotic model of thinking, which departs from it. The quantum semantics is shown to be in accordance with Bion’s model of thinking.

One should consider that the existence of an original all-purpose mental object is convenient for the mind, since such an existence allows it a greater degree of ductility in thinking. Thus, despite the mainly theoretical character of our proposal, it has clear applications in quantum cognition-for in the setting we propose, any internal object of the mind represented as an infinite singleton, is quantized. The introduction of the all-purpose modality guarantees that the object can be evaluated in the way more convenient to the case. Hence, a really effective judgement concerning the contact with reality can be reached: any object is first internally represented as a quantized element, that corresponds to the Freudian theory of representation [14], and then evaluated by the all-purpose modality. The final result is an open-minded judgement, namely the kind of judgement not yet available in A.I., as stressed in the related debate (see [20]). Moreover, discussing abstract quantized objects in the mind rather than concrete quantized objects in the brain opens an interesting path for the future research on the nature of the formal agreement of Freudian theory with Hameroff–Penrose resonance theory [21]. However, we leave this analysis to future work and we thank Stuart Hameroff for a valuable suggestion, from many years ago, of investigating the connections of Bi-logic with quantum logic (personal communication).

We would like to stress how, via quantum cognition, the intriguing foundational issues of quantum mechanics (see [22]) are now directly involved in the issue of cognition too. In the recent years, due to the application of quantum mechanics to communication and information technology in general, the interpretation of some peculiar quantum phenomena has become more and more important outside of physics. The debate on its foundations has spread throughout (see [23,24]) and continues to refer to the original problems, of which the main one is perhaps the separability problem, one of whose forms is the emergence of the entanglement phenomenon [25], which was originally considered as paradoxical with respect to the idea of the “element of reality” [26]. Might the interpretation of quantum mechanics be so intriguing since it requires the awareness of a different cognitive attitude, that a quantum approach to cognition could indeed supply? This paper is in favor of a positive answer to the question that is obtainable by enriching the standard quantum cognitive theory with the affective component derived by psychoanalysis.

## 2. Some Introductory Elements from Freud and Matte Blanco

The foundations of the psychodynamic model of mental apparatus are deeply embedded in Freud’s early neurological works, especially in his book *On Aphasia* [14]. There, he postulated a model of mind–brain relations based on the idea of *mental representation*. Synthetically, mental representation is a sort of psychic delegate of neural changes, and it allows for the establishment of the psychic superstructure of a human being. That is, mental representation can be considered the foundational unit of the psychic order. The subsequent distinction of two different types of mental representations, namely the *thing-* and the *word*-presentations, makes their role a bit clearer. The former is the set of impressions, both sensory and motor ones, related to an object (which can be either internal or external), while the latter is a complex association of sensory and motor elements that define a single word. In Freud’s view, a word *acquires its meaning by being linked to a thing-presentations, and not by reference to the thing itself*. [14] (p. 213) Therefore, this entails that the symbolic function of the mind depends upon the establishment of the associations between thing-presentations and the corresponding word-presentations. This statement was taken as the basis of the clinical practice of psychoanalysis, but its theoretical power has been somewhat underestimated. Additionally, Freud further elaborated this point in his later works, and specified that not only the aforementioned two types of mental representations pertain to different domains of psychic activity, but they also define two different mental systems—namely the conscious and the unconscious:

*The conscious presentation comprises the presentation of the thing plus the presentation of the word belonging to it, while the unconscious presentation is the presentation of the thing alone.*

[27] (p. 201)

It was not until the Chilean psychoanalyst I. Matte Blanco that this aspect of Freudian thought received serious consideration. He reformulated the Freudian conscious-unconscious duality in terms of the underlying logics of these processes, and this is why his theory is known as Bi-logic. According to him, any psychic act is essentially a combination of these two modalities, at various degrees. In *The Unconscious as Infinite Sets* [11], he proposed a reformulation of the Freudian Unconscious in purely logical terms. Namely, he described the Unconscious as a standalone way of thinking and being, denominated *Symmetric Mode* and characterized by two principles:

- the principle of symmetry;
- the principle of generalization.

According to Matte Blanco, *the Unconscious Mode treats the asymmetrical relations as if they were symmetrical* [11] (p. 38). The word symmetry refers to the *sameness* or identity between two things and their fundamental indistinguishability. In particular, since the relation of contradiction is nevertheless a relation, the Unconscious treats opposites as identical. Furthermore, since time can be described as a series of moments where one follows the other, one bizarre consequence of the application of the principle of symmetry is the complete disappearance of time. On the other hand, the principle of generalization reflects the fact that the Unconscious does not deal with individual elements, but only with classes to which they belong. To provide an example, for the child, the mother is not a single, individual person; it is rather a sum of all of the attributes of all members of its defining class—the class of mothers. Therefore, the individual thing is made identical to the class it belongs to. Based on Dedekind’s observation that if a set is equivalent to its part, the set itself is necessarily infinite; Matte Blanco’s formal explanation accounts for the infinite, all-or-nothing character of unconscious processes. Equivalently, Matte Blanco describes the symmetric mode as homogeneous and indivisible, whereas the other mode of B i-logic, termed bivalent, is finite and heterogeneous; namely, it can separate objects. In the following, we shall give a formal logical account for this.

Matte Blanco’s theoretical position therefore defines the Unconscious not in terms of its content, but in terms of its structure. Thus, the idea of a structural Unconscious implies that any model of the mind must include a separate ideation, *a different thought constructing agency* [28]. The introduction of unconscious ideation as an equitable peer of the conscious one also requires a description of its organizing principles. The most notable such description is found in the book *The Interpretation of Dreams* [15], where Freud characterized the Unconscious as akin to the so-called primary process. Namely, while the aforementioned absence of words from the Unconscious remains its most distinctive characteristic, its non-discursive nature is outlined by the following set of principles:

- Displacement;
- Condensation;
- Absence of contradiction;
- Substitution of the external reality with the internal one; and
- Timelessness.

On the other side, the secondary process takes into account the external reality and then it can form rational thinking. In the following section, we introduce infinite singletons, namely those formal elements adopted to discuss how displacement and condensation, namely the peculiar mechanisms of the Unconscious, could be formally represented (see [12] for a discussion). In the present paper, we show that they correspond to thing-presentations, and see how this fact enables us to explore the border between the primary and the secondary process, namely to represent and discuss, from the point of view of a formal logical approach, the role of external reality and its advent in the mental life. This goes together with the analysis of the role of time, and allows us to discover how negation and contradiction can be conceived, and hence see how logic is associated to the advent of the secondary process. The same setting allows us to consider the role of the unrepresented elements in the mental life on the basis of the elements of the Bionian theory, and hence finally characterize the components that can contribute to the formulation of our judgements.

## 3. Infinite Singletons, Open and Closed Presentations and the Corresponding Modelization in Logic

We need to stress that, subscribing to a perspective suggested by both Freud and Matte Blanco, the primitive roots of thinking are unconscious, and hence, the original mode of thinking is infinite. That means that the finite is indeed the negation of infinite, and not the other way around. In a way, language points us in a wrong way, since the kind of infinite we are familiar with—the mathematical infinite—is only a derivative of the original symmetric infinite, and can only be conceived after the finitization of single elements.

Hence, our logical model starts by considering infinite sets. Since our conscious point of view is finitized, let us start from a finite set *V*, given by characterizing its elements, $V_{=}\left\{u_{1},\cdots u_{n}\right\}$, that allows us to count *n* elements in *V*. We now see that the notion of being finite rather than infinite, in logic, is level-sensitive. Considering $V_{=}\left\{u_{1},\cdots u_{n}\right\}$, one is not kept to assume a priori, at the formal, object level, the equivalence:

(1) $$x\in V\equiv x=u_{1}\vee \cdots \vee x=u_{n}$$

If no equivalence of the form (1) is assumed in a logical system, the elements of *V* are uncountable in that logical system, since, picking up a generic element x∈V, there is no way to identify it as one of the *n* elements $\left\{u_{1},\cdots u_{n}\right\}$ in the logical system, and hence, the counting process fails in such a case. In the process of counting, one needs to characterize the elements and separate the elements that have already been considered from the other ones, at any time; hence, one has to assume that the membership relation takes the form (1) in such a case.

As a mathematical example of the failure of (1), one can consider the power set P({1})={∅,{1}}, that is, the set of all propositions modulo logical equivalence. It has cardinality 2 in classical logic since, given any element of P({1}), namely any B⊆{1}, the disjunction B=∅∨B={1} means 1∈B∨1∉B, which is true in classical logic. However, P({1}) is infinite according to intuitionism (see, e.g., [29], p. 6). Considering a subset $B_{U}\subseteq \left\{1\right\}$ such that $1\in B_{U}$ is equivalent to an undecided proposition *U*, $B_{U}\in P\left(\left\{1\right\}\right)$ is not equivalent to $B_{U}=\emptyset \vee B_{U}=\left\{1\right\}$, that is, $1\in B_{U}\vee 1\notin B_{U}$, since the last is U∨¬U, which is not true in intuitionistic logic. Then, being infinite or finite depends on the logical system adopted. In particular, since satisfying an intuitionistic disjunction is harder than satisfying a classical disjunction, more sets are infinite, according to intuitionism.

A second reason for which (1) may not be assumed is that it cannot be expressed; namely, closed terms $u_{i}$ to denote the elements, previously characterized at the metalevel, are not available in the internal language. Actually, the first intuitionistic school, namely Brouwer [10], was very sensitive to the independence of mental objects of language. Such an independence is the key when considering Matte Blanco’s symmetric mode of bi-logic, for in the symmetric mode, every relation is symmetric, since the unconscious can deal with symmetric relations only. Then, since—given a set where one can distinguish two different elements, *a* and *b*—one can put an order: a<b, a set where all relations are symmetric cannot contain two elements recognizable as different, and hence, it must be a singleton. Further, in the symmetric mode, every set is infinite. Hence, our model requires *infinite singletons*. We now see how to conceive them by considering the above definition (1). Let us first consider a singleton given in the standard way, for example, the singleton V={u}. By (1), asserting its finiteness requires assuming

(2) x∈V≡x=u

Now, let us assume that one can form no word to say the object in their mind; namely, no kind of word/label/denotation for the element of *V* is available, so in no way can (2) be given in the mind. Then, *V* would be a good candidate for infinity: it is a kind of symmetric infinity, since it occurs without finitization of single elements. In order to define it as a singleton, without specifying its element, we need to adopt a non-extensional characterization (we recall that an *extensional* definition in logic is a definition giving meaning to a term by specifying its extension, that is, every object that falls under the definition of the term in question). We would like to add that non-extensional views of sets are exploited in our normal reasoning. For example, as in [12], let us consider the sentence “every day of last week was sunny in my town”. In order to find out if it is true, someone who was out of town last week may observe that the ground in their garden is wet and conclude that the sentence is false, without knowing which day it rained, considering the week as a whole. Notice that quantifying on the elements, as in the example, makes it possible.

Infinite singletons are then non-extensionally characterized by the following equivalence, which can characterize a set *V* as a singleton even without assuming the existence of a closed term $\bar{u}$, for which the equivalence $z\in V\equiv z=\bar{u}$ is true:

(3) (∀x∈V)A(x)≡(∃x∈V)A(x)

for every *A*.

Then, infinite singletons come out as non-extensional domains, since no requirement concerning extensionality has to be assumed when adopting quantifiers. Without extensionality, their elements cannot be distinguished one with respect one another: Indeed, considering a non-empty set *V*, namely z∈V, where *z* is a generic element that is not necessarily specified by a closed term, and assuming (3), one has (∀x∈V)x=z≡(∃x∈V)x=z (the case of the empty set will be considered in Section 7.3).

In terms of the Freudian theory, infinite singletons allow us to form thing-presentations, namely the open representations of objects, prior to word-presentations (see the above section). As seen in [12], infinite singletons can be processed by the primary process, in the form of assertions given by the quantification by ∀ or ∃, which is equivalent for them indeed. We would like to observe that this creates, ipso facto, a prelogical environment for the world of infinite singletons. For on one side, it can consider quantification, which is adopted in our conscious reasoning; but on the other, it cannot distinguish between the existential and the universal one. In our mind, infinite singletons are present as unspeakable objects, which only poets can describe by words, for the poetic language can avail of metaphors and other constructions recalling our unconscious constructions [30].

In the standard logical setting, (∀x∈V)A(x) and (∃x∈V)A(x) are closed predicates. However, they are open as long as their domain *V* is open, such as in the case of infinite singletons. When the thing-presentation is closed by a word $\bar{u}$ or a set of words $\left\{{\bar{u}}_{1},\cdots {\bar{u}}_{n}\right\}$, the equivalence $x\in V\equiv x=\bar{u}$ or $x\in V\equiv x={\bar{u}}_{1}\vee \cdots \vee x={\bar{u}}_{n}$, is recognized, and so the infinite singleton *Vunfolds* (in Matte Blanco’s terminology [11]) into the singleton {u}, or into the finite set $\left\{u_{1},\cdots ,u_{n}\right\}$. Then, the corresponding assertions become propositional closed assertions, in particular the closed predicate A(u), in case a finite singleton is recognized. In the other cases, they become $A\left(u_{1}\right)\&\cdots \&A\left(u_{n}\right)$ for the universal quantifier and $A\left(u_{1}\right)\vee \cdots \vee A\left(u_{n}\right)$ for the existential quantifier, since the finitization of infinite singletons allows the ascent of logical duality, as we shall see later.

As an example, let us assume that someone can see that something is flying in the sky, but they cannot attribute a value to the object they see (for example, a seagull). Let us assume that they can reach a thing-presentation of that object, corresponding to an infinite singleton *V*. Then, they can gather the information on the object by quantifying on *V*, and, if A(x) stands for “*x* is flying in the sky”, they can say what they see by the formula (∀x∈V)A(x), or, equivalently, by the formula (∃x∈V)A(x), which is the same on singletons. In order to close the open representation corresponding to the perception of “*x* is flying in the sky”, one has to acquire a closed representation from the external reality, namely, learning the word from a source already able to label the experience. According to Freud ([14]), word-presentations correspond to memory traces of the spoken words; then, as such, they are necessarily acquired from the external reality. Hence, they also include a prescriptive element, which generalizes the experience of being instructed about the name, corresponding to a given experience of a thing that is already acquired in the mind in the form of a thing-presentation. We now see how the prescriptive element can be represented in formal language by a closure process.

## 4. Modal Assertions

The above considerations suggest us to consider non-extensional logical frameworks. Non-extensional logics are usually developed by means of modalities. There are intensional operators for modal attitudes, such as necessity, possibility, belief or knowledge. Modal systems are suited to represent the attitude with respect to contact with reality, prior to expressing a true/false judgement after such a contact. For, as we see below, the truth value can be considered the extension of a sentence, as the reference of an individual name is the extension of a term.

We now see how modal assertions can be derived in our setting, located in between open and closed presentations. Let us assume that there is a collection of infinite singletons that refer to the same object, corresponding to different ways to be in contact with it and inducing different thing-presentations. Then, the value of that object is better grasped, considering the whole collection of infinite singletons for it. Each assertion (∀x∈V)A(x) for each infinite singleton *V* associated to A(x) is characterized by the following definition of quantifier on a domain *D*. In the following, logical constants are defined by means of equations, a technique introduced in basic logic, a system for substructural logics developed in [31]; see [32] for quantifiers:

(4) Γ(−z)⊢(∀x∈D)A(x)≡Γ(−z),z∈D⊢A(z)

where Γ(−z) means that Γ is closed with respect to the free variable *z*. Let us abstract definition (4) with respect to the collection of infinite singletons that refer to the object. Then, one obtains a definition of the form:

(5) □Γ⊢□A≡□Γ⊢A

where □ indicates that a formula is closed with respect to any variable, which refers the value of the object for any thing-presentation of the object. One can prove that the equivalence defines the modal operator of S4, the *necessity* operator [33]. Since in S4, □□A=□A, the operator □ can attribute a sharp yet undefined value to the object. Then, □A is located amid the open/infinite representation created by an infinite singleton *V* and expressed by the formula (∀x∈V)A(x), and the closed word-presentation expressed by the formula A(u).

## 5. Infinite Singletons and Modality in the Spin Model

Infinite singletons were originally conceived in order to model quantum states in first-order logic (see [13,33]). One first considers the set $V=\{t_{1},\cdots ,t_{n}\}$ of the results of the measurement of a quantum particle with respect to a given observable (for the sake of simplicity, one can assume that the observable is discrete). If the sentence “the particle is found in state $s_{i}$ with probability $p_{i}$” is represented by the formula $A(t_{i})$, the mixed state obtained after measurement is described by the conjunction of all possible outcomes, that is, by the formula $A\left(t_{1}\right)\&\cdots \&A\left(t_{n}\right)$. In particular, when the state is sharp (one possible outcome *u* only), it is described by the closed predicate A(u). Then, let us consider the open formula A(z), where the variable *z* stands for the unknown state of the particle prior to measurement. In logic, one can represent the effect of measurement by the substitution of the free variable *z* for the state by the possible outcomes $t_{i}$. Like the quantum measurement in physics, the substitution of a variable by a closed term is an irreversible transition in logic, unless the membership z∈V is assumed equivalent to the disjunction of its possibilities: $z=t_{1}\vee \cdots \vee z=t_{n}$ (equivalence 1). In such a case, one could re-derive the open formula A(z) from the conjunction $A\left(t_{1}\right)\&\cdots \&A\left(t_{n}\right)$ by the laws of equality (see [13]). Then, let us assume that the membership relation z∈V is not equivalent to the disjunction $z=t_{1}\vee \cdots \vee z=t_{n}$. This means that one is not identifying the generic result *z* with one of its possibilities, and hence that one is referring to the state prior to measurement. Then, quantified formulae over infinite singletons (∀x∈V)A(x) can assert the state of the particle, with respect to the given observable.

In particular, one can consider the spin observable. Then, measuring a particle with respect to a given direction, two distinct outcomes: ↑ “up” and ↓ “down” with respect to that direction are possible. Prior to measurement, the two cases are indistinguishable, and an infinite singleton is formed. Considering the collection given by the measurement of the spin for each direction, one can abstract with respect to the direction, and applying definition (5), find a formula □A that is closed with respect to each variable for each direction, obtaining the modal operator □ of S4, as seen in the above Section 4. The equality □□A=□A for every *A*, that holds in S4, shows that □ can be interpreted as an general projector, the modal projector, in an abstract space (see [33]).

## 6. Infinite and Finite Interpretation of the Modality in the Quantum Model and in Psychoanalytic Theory

Once we have produced the modal projector □, we call *T* the infinite singleton resulting from the application of the projector □ in the abstract space associated with the measurement in all possible directions. *T*, in turn, is the domain of □, interpreted as a higher-level quantifier. Equation (5) is itself an equation of the form (4), and so the modal formula □A can be interpreted as quantifier on the domain *T*: □A=(∀x∈T)A(x). The domain *T* interprets the infinite/open content of □A. We are reminded that a single infinite singleton corresponds to the formalization of the Freudian thing-presentation. A subsequent development of the theory was produced by the British psychiatrist and psychoanalyst Wilfred Bion, who defined the theoretical construct of the *mental container*. According to this theory, a concept can be derived when the mental container is associated to a *content* derived from experience. In his theory, the container is an open and abstract domain that can never be saturated by the content. In our formal model, the Bionian construct of the container is represented by the domain *T*. It is important to note that according to Bion, the container is a timeless a priori of experience, and therefore, it is an abstract function that makes experience possible. In fact, experience takes place via the container–content relationship which develops in time. The container in touch with the experience is no more the a priori, since it is modified by its link with the content; Bion therefore calls it “Container^*n*^”. Below, one can find some quotations from [19], illustrating the role of the container, and therefore providing the psychoanalytic basis for our model. Quotations from Chapter 27 concern the positive link, labeled “*K*” (from Knowledge); quotations from Chapter 28 concern the negative link, labeled “−K”.

*11. The activity that I have here described as shared by two individuals becomes introjected by the infant so that the Container-Contained apparatus becomes installed in the infant as part of the apparatus of alpha-function. A model is provided by the idea of the infant who explores an object by putting it into his mouth. What talking was originally done by the mother, possibly a rudimentary designatory function, is replaced by the infant’s own baby talk.*

*12. Using 11 above as a model from which to abstract a theory to represent the realization of the development of thoughts, I propose the following terms:*

*(a) Pre-conception. This term represents a state of expectation. The term is the counterpart of a variable in mathematical logic or an unknown in mathematics. It has the quality that Kant ascribes to an empty thought in that it can be thought but cannot be known.*

*(b) Conception. Conception is that which results when a pre-conception mates with the appropriate sense impressions. I have used a phrase in which the implied model is obvious. The abstraction from the relationship of preconception to sense impressions is Container to Contained (NOT Contained to Container).*

(ch. 27, points n. 11 and 12)

Then, the abstract domain *T* is the container, which is a preconception that allows the mind to get in contact with a given experience. The container allows us *to learn from the experience of reality*, by developing Container${}^{n}$; in his words:

*19. Learning depends on the capacity for the Container${}^{n}$ to remain integrated and yet lose rigidity. This is the foundation of the state of mind of the individual who can retain his knowledge and experience and yet be prepared to reconstrue past experiences in a manner that enables him to be receptive of a new idea. (⋯)*

(ch. 27, point n. 19)

In the following words, one can read that repetition in time results into a particular kind of abstraction, termed *commensal abstraction*, which allows us to learn. The *container–contained interaction* is particularized by the positive link *K*, which he terms the commensal link.

*13. To summarize. The relationship between mother and infant described by Melanie Klein as projective identification is internalized to form an apparatus for regulation of a preconception with the sense data of the appropriate realization. This apparatus is represented by a model: the mating of pre-conception with sense-impressions to produce a conception. The model is in turn represented by Contained-Container.*

*14. The repetition of mating of pre-conception and sense data, that results in commensal abstraction, promotes growth in Contained and Container. That is to say the capacity for taking in sense impressions develops together with the capacity for awareness of sense data. (⋯)*

(ch. 27, points n. 13 and 14)

Building on his clinical experience, Bion produces an attempt to formalize the difficulties that can arise from getting in touch with reality. He therefore models a link that is contrary to *K*, labeled −K, the *parasitic link*, corresponding to negative attitude. It is attributed to envy. The role of envy as a substitute of the positive relation is illustrated as follows:

*4. Inevitably one wonders at various points in the investigation why such a phenomenon as that represented by −K should exist. The answer to that question must be sought in psycho-analytic work with individual patients. I shall consider one factor only—Envy. By this term I mean the phenomenon described by Melanie Klein in Envy and Gratitude.*

*5. I have described the role of projective identification in K as a commensal relationship between Container and Contained. In −K, (⋯), the relationship of Container to Contained is represented by Container + Contained where + can be replaced by Envy. Using this formulation to represent infant and breast (to resort to less abstract signs) and using as a model an emotional situation in which the infant feels fear that it is dying, the model I construct is as follows: the infant splits off and projects its feelings of fear into the breast together with envy and hate of the undisturbed breast. Envy precludes a commensal relationship. The breast in K would moderate the fear component in the fear of dying that had been projected into it and the infant in due course would re-introject a now tolerable and consequently growth-stimulating part of its personality. In −K the breast is felt enviously to remove the good or valuable element in the fear of dying and force the worthless residue back into the infant. The infant who started with a fear he was dying ends up by containing a nameless dread.*

(ch. 28, points n. 4 and 5)

In the following words of Bion, we read a general abstract interpretation of the framework determined by −K:

*13. ⋯ In practice it means that the patient feels surrounded not so much by real objects, things-in-themselves, but, by bizarre objects that are real only in that they are the residue of thoughts and conceptions that have been stripped of their meaning and ejected.*

*14. The relationship of K to −K can be epitomized by saying that in K particularization and concretization of the abstract and general is possible, but in −K it is not because the abstract and general, in so far as they exist, are felt to become things-in-themselves. Conversely in K the particular can be generalized and made abstract, but in −K the particular becomes denuded of any quality it has; denudation not abstraction is the end product.*

(ch. 28, points n. 13 and 14)

We would like to mention that a different theorization of the same phenomenology concerning contact with reality was described by the French psychoanalyst Piera Aulagnier in *The Violence of Interpretation: from Pictogram to Statement* (see [8]).

## 7. Splitting the Modality

We have seen above that Bion recognizes that the experience of reality entails developing a container (that he labels Container${}^{n}$; see (ch. 27 “*K*”, point n. 19), quoted above). Now, let us assume that time is in the process of abstraction that leads to Equation (5), and the definition of *T* can be recognized. This happens due to the attitude with respect to the external reality, when inner and external reality have to come in touch. Then, in physics, an initial condition of measurement for that process can be assumed, considering the Schroedinger picture as in [33]. That is, a description of the evolution of the state with respect to a fixed observable (see [34] p. 125). This allows to discuss the line developed by Bion and Freud in terms of initialization.

In the spin model, the finitization of *T* can be obtained considering the temporal evolution of the spin, induced by a temporal parameter (see [33]). We have a split of the possibilities: one for each direction *d* of the spin. Rather than (5), we have, in general, an equation where an initial condition $\sigma_{d}$ is specified in the assumptions and where a corresponding operator ${□}_{d}$ is obtained:

(6) $$□\Gamma ⊢{□}_{d}A\;\equiv \;□\Gamma ,\sigma_{d}⊢A$$

Let us consider now the three orthogonal cases, $\sigma_{z},\sigma_{x},\sigma_{y}$, and see that they give rise to a positive, negative and irreal case, respectively, which finds its psychoanalytic interpretation. The first two cases, positive and negative, as with the spin model, have also been discussed in [33].

### 7.1. Positive and Negative Case

The positive case, in Bion, is given by the commensal link, which is an agreement between container and contained (see Bion, [19], ch. 27 “*K*”, points n. 11-14, quoted above). It corresponds to the acceptance of the representation of reality one has achieved, by means of the formed infinite singletons. Then, the modality □ is kept, but the infinite singleton *T* is reduced to a single positive element *p*. In the quantum model, this happens when the observable of the initial condition in Equation (6) is the same as the preparation, *i.e.*, the direction is along the *z* axis. Then its matrix is the real Pauli matrix $\sigma_{Z}$. $\sigma_{Z}$ is the real combination of the two projectors $P_{↑}$ and $P_{↓}$ onto the two elements of the basis ↑ and ↓ (with respect to the *z* direction): $\sigma_{Z}=P_{↑}-P_{↓}$. Then, one derives the finite version of □; namely, the domain *T* reduces to the positive element *p*, representing the unique abstract direction of the spin, on which the state is projected. Then, □ has no open character any more. The value of □A is assertive: *A* is necessary, then it is true. In S4, one has that □A→A is true; hence, assuming □A, one derives *A*. When a word-presentation of the object is reached, it is asserted: A(u) (see [33]).

The negative case. This is the case developed by Freud in his paper *Negation* [17]. It corresponds to the rejection of the representation of reality one has achieved, which according to Freud means indeed its repression. In the quantum model, it is obtained when the observable is the spin along the *x* direction, orthogonal to the direction *z* of the preparation, and the observable is described by the other real Pauli matrix, $\sigma_{X}$. It is the real combination of the two antiprojectors $Q_{1}$ and $Q_{2}$: $\sigma_{X}=Q_{1}+Q_{2}$. Then, solving Equation (6) with d=x, one finds the abstract antiprojector, which, applied to an element of the basis, finds out “the other element”, namely a negative element representing “the other” with respect to the result of the projector. It is a negative element *n* extracted from *T*. Then, the equation with the $\sigma_{x}$ assumption defines a negative modality ${□}_{n}$, an operator that makes us able to assert the negation of the representation derived from the contact with reality. The infinite formula (∀x∈T)A(x) is reduced to A(n), which is identified with the negative modal formula ${□}_{n}A$. One can interpret it as an explicit negation ∼, putting, on the modal formulae □A, $∼□A\equiv {□}_{n}A$. When a word-presentation *u* of the object is reached, it is negated: ∼A(u) (see [33]). In particular, since the logical constant for falsum is interpretable in the spin model, the negation ∼ so defined satisfies the non-contradiction law (for the interpretation and the proof, see [33]). Then, negation is introduced as a characterizing feature of the secondary process. The negative character corresponds to the Freudian conception of negation as the intellectual counterpart of repression. For in the spin model, repression can be interpreted as a switch from the original basis of eigenvectors along the *z* direction, which makes word-presentation possible, to the orthogonal basis whose eigenvectors are given by $\sigma_{x}$ (see [33]).

### 7.2. Irreal Case

The irreal case corresponds to the parasitic link in Bion, labeled −K in the quotations above. It is a more primitive case, closer to the original open/indefinite view of □, it represents the opposite: it corresponds to the failure of contact with reality caused by a failure of representation. Following Bion, the link is negative; this means that the preconception is abolished: no infinite singleton is created, and hence, the domain *T* is empty (from an extensional point of view). In the unconscious, at the intensional level, such an attitude produces what Bion has termed as the bizarre elements (see [19], ch. 28 “−K”, points n. 13 and 14 quoted above). These are the impossible elements of the empty set, prior to its extensional interpretation. The modality □ is converted into an impossibility: let us write it □, meaning “the contrary of being necessary”.

We now look for the third element *e* of *T* (analogous to *p* and *n*) to characterize □A as A(e). In the quantum model, it is given considering the observable $\sigma_{y}$ in (6), and hence the third Pauli matrix $\sigma_{Y}$. $\sigma_{Y}$ has imaginary entries:

$$\sigma_{Y}=\left(\begin{array}{cc} 0 & -i\\ i & 0 \end{array}\right)$$

Like $\sigma_{X}$, it can be read as a superposition of the two antiprojectors, namely $Q_{1}=\left(\begin{array}{cc} 0 & 0\\ 1 & 0 \end{array}\right)$ and $Q_{2}=\left(\begin{array}{cc} 0 & 1\\ 0 & 0 \end{array}\right)$, but with complex coefficients:

$$\sigma_{Y}=iQ_{1}+-iQ_{2}.$$

Then, unlike $\sigma_{X}$, $\sigma_{Y}$ fails to provide a real negation, and hence, it fails to provide a representation that has to do with the external reality—only internal representations are created. Particularly, its role is better grasped after an analysis of impossibility.

### 7.3. Logical Considerations on Impossibility

In order to better focus on the irreal case, let us go back to infinite singletons and clarify the opposite role of □, impossibility, with respect to □, *i.e.*, the necessity. Equivalence (3): (∀x∈V)A(x)≡(∃x∈V)A(x), characterizing singletons, says that one of the two quantifiers is enough for singletons. We adopt the universal one, since it gives account for the collapse from infinite to finite, described by the logical consequence (∀x∈V)A(x)⊢A(u). The last, assuming u∈V, is derived by substitution of the closed term *u* for the variable *z* in (∀x∈V)A(x),z∈V⊢A(z). Actually, prior to the contact with reality, one should better say that there is a unique quantifier, say, the “symmetric quantifier”. However, one needs to see how the symmetric quantifier splits into two due to contact with reality, so referring to the existing formal one is better, at least so far. Equivalence (3) is not true in all the other cases; however, in classical logic, one can characterize a set *E* as empty by assuming the following negated form for a given *A*:

(7) (∀x∈E)A(x)≡∼(∃x∈E)A(x)

Actually, one could adopt the above (7) as an intensional characterization of the empty set. By comparing (3) and (7), one could say that the first gives the attitude to include and the second to exclude. We show how one could consider the last as the privileged way of the existential quantifier. The usual definition of existential quantifier considers implicitly a conjunction, since (∃x∈D)A(x) means ∃x(x∈D&A(x)). Then, the equation to introduce it has the form (see [32]):

(8) (∃x∈D)⊢Γ(−z)≡A(z),z∈D⊢Γ(−z)

which can be rewritten:

(9) (∃x∈D)⊢Γ(−z)≡A(z)⊢z∉D,Γ(−z)

This last one is the symmetric of Equation (4), with respect to the consequence sign ⊢ (for the formal definition of a symmetric equation, see [31]), but it contains the membership predicate in the negated form. Namely, in order to introduce the universal quantifier, one has to consider an inclusion, whereas for the existential one, an exclusion is taken into consideration.

Going on, one can consider the abstraction of (9) with respect to the domain, and define the modality ⋄, “possible”, dual of □, “necessary”, by writing the symmetric of definition (5):

(10) ⋄A⊢⋄Γ≡A⊢⋄Γ

In turn, since in our interpretation, ⋄ is the abstract existential quantifier, it hides its own domain *N*, having the dual attitude of the container *T*, which is the exclusion attitude. This means that, *extensionally*, *N* is the empty set. However, *intensionally*, at the symmetric level, we can conceive *N* as *T* inhabited by an element *e* that can empty it. This is consistent with the destroying role of envy, described in the quotation from [19] (ch. 28 “−K”, points n. 4 and 5, reported above).

We recall that in the symmetric mode of Matte Blanco [11], where everything is infinite, the generalization principle does not allow for the exclusion of an element from a set. So, one could consider the above symmetric Equation (10) defining the dual modality ⋄ as the rational way out for impossibility. This, in turn, allows us to form the idea of impossibility by negation in our conscious reasoning, since “impossible” is the negation of “possible”. Formally, ⋄A⊢ means ⊢∼⋄A, “*A* is not possible”.

In a different conscious interpretation, the idea of impossibility can be given as the contrary of necessity, notably as the necessity of a negation. Namely, □A should assume the form □¬A, where ¬ is a negation. This implies that the “inner negative attitude” ¬ (a prior to the representation given by □) should be induced. It is not the conscious negation ∼, defined previously as the rejection of a reality, that has been represented anyway. At the conscious level, it disappears: one identifies □¬A with ∼⋄A, that is, the negation of possibility, where ∼ is the conscious negation. Hence, the inner negative attitude is absorbed by the usual negation, as defined in the classical logic. Quoting Freud in *Negation* [17]:

*The general wish to negate, the negativism which is displayed by some psychotics, is probably to be regarded as a sign of a defusion of instincts that has taken place through a withdrawal of the libidinal components. But the performance of the function of judgement is not made possible until the creation of the symbol of negation has endowed thinking with a first measure of freedom from the consequences of repression and, with it, from the compulsion of the pleasure principle.*

As a final remark, we would like to go back to the hypothesis about $\sigma_{Y}$ in the spin model. Namely, the inner negative attitude is a nonreal superposition of the two antiprojectors, due to the structure of $\sigma_{Y}$. In this setting, the commutation relations can tell us something about what happens “outside”, after measurement. That is, assuming that contact with reality takes place. Since $\sigma_{Y}=\frac{i}{2}\left[\sigma_{Z},\sigma_{X}\right]$, the Pauli matrix $\sigma_{Y}$ is then conceivable from the two others. Further analysis of the correspondence of this fact with the definability of “impossible” by duality, in classical logic, is left to further work.

## 8. The Components of Judgements

Let us consider now Equation (6) for a generic direction *d* of the spin. As is well known, a spin observable $\hat{O}$ is described by a 2×2 complex self-adjoint matrix, that is:

$$\hat{O}=\left(\begin{array}{cc} r_{1} & \beta_{X}-\beta_{Y}\\ \beta_{X}+\beta_{Y} & r_{2} \end{array}\right)$$

It corresponds to the real linear combination:

$$\hat{O}=\alpha I+\beta_{Z}\sigma_{Z}+\beta_{X}\sigma_{X}+\beta_{Y}\sigma_{Y}$$

of the elements of the orthonormal basis given by the three Pauli matrices and the identity matrix: $I=\left(\begin{array}{cc} 1 & 0\\ 0 & 1 \end{array}\right)$, where $\alpha +\beta_{Z}=r_{1}$ and $\alpha -\beta_{Z}=r_{2}$.

The linear combination is often written (see, *e.g.*, [34], p. 223):

(11) $$\hat{O}=\alpha I+\bar{\beta }\cdot \hat{\sigma }$$

where $\bar{\beta }\cdot \hat{\sigma }$ is the scalar product of the real vector $\bar{\beta }=\left(\beta_{X},\beta_{Y},\beta_{Z}\right)$ and of $\hat{\sigma }=\left(\sigma_{X},\sigma_{Y},\sigma_{Z}\right)$. In physics, $\bar{\beta }$ is a non-null vector, since the observable must be nontrivial, that is, $\hat{O}=\sigma_{d}$ for some direction *d*. Let us assume that α≠0 and $\bar{\beta }=\bar{0}$. Since any pair of orthogonal vectors, for any direction *d*, is a pair of eigenvalues for the identity, initializing an equation like (6), with the identity rather than the observable corresponding to some fixed direction, means that no initialization is actually given. While such an initialization cannot have anything to do with what an experimenter can do in a physics laboratory, it has something to do with the representation that the same experimenter can conceive about what they are doing (and in general, with our thinking as human beings). Then α≠0 and $\bar{\beta }=\bar{0}$ yields the neutral case: the timeless definition of the modality (5) that corresponds to the whole container *T*, and hence to the infinite case, as seen above.

In general, the initialization of the Schroedinger picture with an observable, as in (6), can produce an attitude with respect to reality. Hence, a generic judgement includes both an infinite attitude, given by the α coefficient of the observable; and a finite one, given by the $\bar{\beta }$ vector. It includes the positive, negative and irreal attitude towards the contact with reality in different proportions. This corresponds to the idea at the basis of Bion’s theorization recalled above, as well as Matte Blanco’s bi-logic—indeed, following Matte Blanco, our thinking requires both a symmetric/infinite mode and a finite/bivalent mode, otherwise no thinking is possible [35].

### Concluding Remarks on the above Interpretation of Judgements in Bion’s Theory

In a way analogous to Matte Blanco in his *Thinking, Feeling and Being* ([35]), in the last part of his life Bion has also figured the idea of *O* (from “object”) to mean the totality of the contact with reality. This is known, in existential terms, in his theory of mental transformations [36]. *O* indeed stands for the unknowable ultimate reality, which cannot be described but only lived-you cannot “know *O*”, you must “become *O*”. In particular, he posed the need to overcome the level of knowledge at the heart of mental life, known as *transformation from K to O*. In a homonymous essay, first published in 1965 [36], Bion proposed *to extend the significance of O to cover the domain of reality and “becoming”. Transformations in O contrast with other transformations in that the former are related to growth in becoming while the latter to growth in “knowing about growth”.* ([36], p.156). Transformations in *O* are in strict correspondence with transformations that must occur during the psychoanalytic work, as *Resistance to an interpretation is resistance to change from K to O.* ([36], p.159). We would like to stress that the composition of *O* in our model resumes and combines at an abstract level all possible ways in which mental processes can occur with respect to inner and external reality; it therefore necessarily transcends the level of “descriptive knowledge” in a direction that is clearly reminiscent of the transformation from *K* to *O*. In our proposed theory of judgement, where the spin model is allowed to be summarized by Equation (11), the real coefficient α≠0 in the equation itself allows us to keep into account the infinitary aspect of the mental life. Significantly, this is different from what the cognitive theories, based on explicit cognitive representations, can perform (see [1]). Namely, a judgement is enabled to include the whole mental life. Some quotes from Bion that can better illustrate his thinking are reported below:

*Transformations in O contrast with other transformations in that the former are related to growth in becoming and the latter to growth in “knowing about growth”. Resistance to an interpretation is therefore resistance to the change from K to O. Such a change is “of particular concern to the analyst in his function of aiding maturation of the personalities of his patients.*

([36], p.156)

*O represents the summation of the energy mass of infinite information (emotional and otherwise) that is always evolving. Correspondingly, we unconsciously experience a destiny to seek it, prompted by our truth drive to keep this rendezvous, or to avoid it out of anxious premonition.*

*The analyst must focus his attention on O, the unknown and unknowable ⋯ He cannot identify with it. He must be it ⋯ It stands for the absolute truth in and of any object; it is assumed that this cannot be known by any human being; it can be known about, its presence can be recognized and felt, but cannot be known. It is possible to be at one with it ⋯ No psychoanalytic discovery is possible without recognition of its existence, at-one-ment with it and evolution. The religious mystics have probably approximated most closely to expression of experience of it ⋯ Its existence is as essential to science as to religion.*

(Bion, quoted in [37], pp. 1095–1096)

And Grotstein ([37]) observes:

*I believe that Bion became aware that psychoanalytic ideas had become a prisoner to the domain of the senses and its sense (image)-derived language—the ‘language of substitution’ (Bion, 1970, p. 126). The latter constituted a domain that had accommodated linear science, whose proper object, Bion felt, was inanimate objects but which was blind to human experience and to the existence of an ultra-sensual world beyond the third dimension—until the arrival of the theories of relativity and uncertainty (pp. 6–25). Animate objects (humans) defied the confines of linear science, he believed (1963, p. 11). He thus set out to formulate the ‘Language of Achievement’ (1970, p. 126), a language that derived from intuitive explorations of the ultra-sensual internal and external worlds in terms of emergent emotions, and is responsive to such ideas as ‘transcience’ (Kitayama, 1998) and ‘uncertainty’ (Heisenberg, 1958). His mission was to shift psychoanalysis from inanimate to animate science and to pull it from deterministic certainty to relativistic uncertainty, which, since Einstein and Heisenberg, is closer to our current concepts of cosmic and human truth.*

([37], p. 1084)

## Data Availability

Not applicable.
